# Postural and Balance Disorders in Patients with Parkinson's Disease: A Prospective Open-Label Feasibility Study with Two Months of Action Observation Treatment

**DOI:** 10.1155/2015/902738

**Published:** 2015-12-22

**Authors:** Andrea Santamato, Maurizio Ranieri, Nicoletta Cinone, Lucia Anna Stuppiello, Giovanni Valeno, Jula Laura De Sanctis, Francesca Fortunato, Vincenzo Solfrizzi, Antonio Greco, Davide Seripa, Francesco Panza

**Affiliations:** ^1^Physical Medicine and Rehabilitation Section, “OORR Hospital”, University of Foggia, 71100 Foggia, Italy; ^2^Sector of Hygiene, Department of Medical and Surgical Sciences, University of Foggia, 71100 Foggia, Italy; ^3^Geriatric Medicine-Memory Unit and Rare Disease Centre, University of Bari Aldo Moro, 70124 Bari, Italy; ^4^Geriatric Unit & Laboratory of Gerontology and Geriatrics, Department of Medical Sciences, IRCCS “Casa Sollievo della Sofferenza”, San Giovanni Rotondo, 71013 Foggia, Italy; ^5^Neurodegenerative Disease Unit, Department of Basic Medicine, Neuroscience, and Sense Organs, University of Bari Aldo Moro, 70124 Bari, Italy; ^6^Department of Clinical Research in Neurology, University of Bari Aldo Moro, “Pia Fondazione Cardinale G. Panico”, Tricase, 73039 Lecce, Italy

## Abstract

Action observation treatment has been proposed as therapeutic option in rehabilitation of patients affected by Parkinson's disease (PD) to improve freezing of gait episodes. The purpose of this prospective open-label feasibility study was to evaluate the impact of 8-week action observation training (video-therapy) for the treatment of postural instability and balance impairment in PD patients. Fifteen PD patients aged under 80 years with scores of 1 to 3 on the Hoehn and Yahr staging and without evidence of freezing of gait were recruited. They underwent 24 sessions of video-therapy training based on carefully watching video clips on motor tasks linked to balance, subsequently performing the same observed movements. No statistically significant differences were observed in the identified outcome measures with the Berg Balance Scale and the Activities-Specific Balance Confidence Scale after two months of follow-up. In the present study, a short course of action observation treatment seems to be not effective in reducing balance impairments and postural instability in patients affected by mild to moderate PD. Further studies with larger samples, longer follow-up period, and standardized protocols of action observation treatment are needed to investigate the effects of this rehabilitation technique in the management of postural and balance disorders of PD patients.

## 1. Introduction

Postural instability is one of the cardinal manifestations of Parkinson's disease (PD) and becomes a clinical concern in the middle-later stages, usually occurring after the onset of other clinical features [1]. Patients with PD have decreased stability during both static and dynamic motor tasks and the risk of falling represents a serious and disabling issue that affects daily life and personal autonomy and generally may not respond to dopaminergic treatment. Up to 40% of PD patients with postural instability have multiple falls that predispose to injury, including wrist and hip fractures and the need for medical care [2, 3]. In addition, social isolation occurs because of the fear of walking. The pathophysiology for postural instability is still uncertain; however, factors such as the impairment or loss of postural reflexes, disturbance in central sensory processing, inflexibility of postural reflexes, postural deformities, interactions of akinesia, bradykinesia, rigidity, and freezing of gait can contribute to imbalance in PD [4, 5].

The current mainstay of managing postural instability remains conventional physiotherapy (stretching, aerobic training, relaxation and muscle activation, and treadmill walking), although new strategies have emerged and may be used in rehabilitative settings in conjunction with pharmacological treatment. Based on the evidence of the involvement of the mirror neuron system (MNS) in the process of motor learning, a role of action observation treatment (AOT) in the field of neurorehabilitation in PD patients has been assumed. Observation of action performed by others may activate in an observer the same neural network as when he/she actually performs the same action [6]. These observations suggest that there is a common thread between observation and execution of movement through internalization of temporal sequences by the observer and activation of the same cortical motor areas. This assumption represents the neurophysiological rationale for AOT in patients suffering from neurological diseases. AOT (via video) has also been successfully incorporated into clinical stroke rehabilitation programmes, significantly improving motor function, more than physical therapy alone [7, 8], as well as in postsurgical orthopedic patients [9]. The subsequent repetitive execution of the observed actions transmitted via video, generally concerning daily life movements, is the way in which the activation of MNS can be stimulated. Promising results obtained by recent studies showed the improvement of freezing of gait episodes and then quality of life in PD patients undergoing video-therapy sessions [10]. To date, to the best of our knowledge, no study has assessed the role of AOT in the treatment of postural instability. In particular, our attention has been given to the assessment of postural instability and balance disorders, as measured by functional scales, but excluding patients with freezing of gait, which apparently is the only variable that seems to have changed in previous studies. Therefore, the purpose of the present prospective open-label feasibility study was to evaluate the effectiveness of AOT in improving postural and balance disorders and secondly to assess whether AOT could have a positive impact on self-confidence in activities of daily life and risk of falls.

## 2. Methods

The present study is a prospective open-label feasibility study conducted according to the World Medical Association's 2008 Declaration of Helsinki and the guidelines for Good Clinical Practice. This study was approved by the Institutional Review Board of the University of Foggia, Foggia, Italy. Consecutive outpatients with a clinical diagnosis of idiopathic PD according to UK Brain Bank diagnostic criteria [11], attending the Department of Physical Medicine and Rehabilitation, University of Foggia, Italy, from January 2014 to March 2015, were invited to participate and were screened for study eligibility. The patients included in the present study had the following: age of < 80 years, time from PD diagnosis of ≤ 10 years, diagnosis of idiopathic PD made by a senior neurologist, Hoehn & Yahr (H&Y) stage ≤ 3 [12], Functional Ambulation Category (FAC) ≥ 4 [13], stable medication regime for the month prior to and for 2 months of the study period, and any type of rehabilitation in three months prior to and during the study protocol. Exclusion criteria were as follows: vascular and iatrogenic parkinsonism; vestibular dysfunction, cardiovascular, and musculoskeletal problems that might affect balance; Pisa Syndrome; severe visual disturbance; cognitive impairment that could have limited the adherence to treatment, in particular patients with a Minimental State Examination (MMSE) score < 24 [14]; severe dyskinesias or “on-off” fluctuations; and therapies involving cueing strategies or other exercise activities. Subjects with freezing of gait, identified using Freezing of Gait Questionnaire (FOG-Q) item 3 [15], were also excluded from the study. Furthermore, they were asked to make rapid 360° narrow turns from standstill, on the spot and in both directions as a further test to objectively unmask freezing of gait [16]. A total of 15 participants (9 females, 6 males) fulfilling inclusion criteria were enrolled and, after a complete description of the protocol, provided informed written consent to participate in the study.

### 2.1. Procedures

Participants underwent an 8-week rehabilitation programme for 3 times a week, and the treatment was conducted under the supervision of an experienced physiotherapist. Each therapy session took place in a bright and not furnished room to avoid elements that could have an impact on the attention of the patients. The total number of training sessions per subject was 24. Patients were sitting in a comfortable chair and monitor screen (25 inches) was placed 100 cm distance in front of them. They were instructed to carefully watch the videos projected concerning motor tasks and motor sequences linked to balance. The movements were recorded from the front, side, and rear to ensure that patient understood the correct execution in the three dimensions. Two different series of four videos have been shown in the first month of treatment and in the last month. This choice was motivated by the need to both perform motor actions increasingly complex and obtain a greater adherence by the patients. The duration of each video was 1.50′; then, it was repeated two more times with the last one in slow motion (2′), so that the total duration of the action seen was 5 minutes. At the end of each video, patients were requested to perform the observed action for other 5 minutes, with the therapist that constantly encouraged them to perform to their full potential. Every session of AOT and individual rehabilitation lasted 40 minutes.  Table 1 shows the contents of the videos shown for AOT. During the study, participants were instructed to take their Parkinson's disease medications regularly and were trained during the ON phase, within 2 hours of the last dose.

### 2.2. Primary Outcomes Measures

Patients were evaluated at baseline (*t*_0_) and after the end of rehabilitative treatment protocol lasting 8 weeks (*t*_1_) by the same investigator. A battery of clinical tests, including primary and secondary outcome measures, was used. The evaluation was performed at the same time in the morning, during ON condition (<2 hours after the intake of the dopaminergic medication). Among primary outcome measures, Berg Balance Scale (BBS) is a 14-item (0–4 points per task; high = best performance) validated scale that evaluates balance abilities during sitting, standing, and positional changes. Total scores are indicative of overall balance abilities, with a score of 0 to 20 indicating high fall risk; a score of 21 to 40 indicating medium fall risk; and a score of 41 to 56 indicating low fall risk [17]. A score of 43.5 or below suggests risk of falls [18]. The Activities-Specific Balance Confidence Scale (ABC-16) was administered to investigate the self-perceived level of balance confidence while performing 16 daily living activities rated 0 to 100 each. Patients with a score below 75.6 are at risk for falls [18].

### 2.3. Secondary Outcomes Measures

These outcome measures included Unified Parkinson's Disease Rating Scale, part III (UPDRS III) [19], 10-Meter Walk Test (10MWT) [20], and the Timed Up and Go Test (TUG) [21]. The Unified Parkinson's Disease Rating Scale (UPDRS) with four sections (I: Nonmotor Experiences of Daily Living; II: Motor Experiences of Daily Living; III: Motor Examination; and IV: Motor Complications) is scored from 0 to 199 (199 represents the worst disability and 0 represents no disability). Part III has been used as secondary outcome (score ranges from 0 to 108). Items include rest tremor, action tremor, facial expressions, rigidity, bradykinesia, gait, and posture [19]. The 10MWT measures gait speed by the time required to walk 10 meters. The Timed Up and Go Test evaluates functional balance and basic mobility skills by measuring seconds when subject is asked to rise from sitting, walk 3 meters, return, and sit down [20]. Proposed cut-off score for prediction of falls in Pd is 11.5 seconds [21].

### 2.4. Statistical Analysis

All variables and difference between baseline (*t*_0_) and posttreatment outcome measure scores (*t*_1_) were expressed as mean (95% confidence intervals (CIs)) and were compared using the Kruskal-Wallis test. The level of statistical significance was set as *p* < 0.05. Data analyses were performed using STATA MP 10.1.

## 3. Results

The PD patients aged between 60 and 77 years (mean = 68.9 ± 4.7) had a mean disease duration of 3.5 ± 1.9 months. The demographical and clinical characteristics of the 15 PD patients enrolled in the present study are shown in Table 2: 60% (n. 9) of the patients enrolled in the study were female.  Table 3 showed primary (BBS and ABC-16) and secondary (UPDRS III, 10MWT, and TUG) outcome measure scores at baseline (*t*_0_) and after 8 weeks of treatment (*t*_1_), with score differences along with 95% CIs. No statistically significant differences were observed in all primary and secondary outcome measures used in the present study.

## 4. Discussion

Imbalance and postural instability are among the most disabling issues of PD, still partly misunderstood. In the present prospective open-label feasibility study, we aimed to demonstrate the effect of AOT for postural instability and balance disorders in PD patients, as well as occurring of freezing of gait in these patients [10]. AOT is an innovative rehabilitative technique that has been successfully used especially for rehabilitation of stroke patients [7, 8]. Unfortunately, the present findings did not suggest an impact of this technique not effective in reducing balance impairments and postural instability in patients with mild to moderate PD.

Several studies and review articles focused on efficacy of AOT in PD patients [10, 22–25]. AOT combined with the repetition of the observed actions has a positive effect in terms of retention of information but only when observed movements are congruent with practiced movements. The observation of video just performed and the repetition of sequences of movements as occurred in AOT make it possible to stimulate the MNS involving basal ganglia. In fact, previous studies showed the detection of changes in local fields potentials (LFP) recorded from the subthalamic nucleus in PD patients during the observation of a movement performed by another person, whereas no LFP signals were detected during the observation of a static image. These changes were present in both “off” and “on” PD motor states and were similar to those observed during movement execution [26, 27]. These results can explain the effectiveness for the rehabilitation of bradykinesia or motor impairment in PD.

In particular, Pelosin and colleagues have shown that AOT has a positive additional effect on recovery of walking ability in PD patients with freezing of gait [10]. More specifically, the subjects enrolled in the study underwent a 60-minute physical therapy training for 3 sessions/week for 4 weeks. A group of PD patients watched 6 video clips showing strategies useful in circumventing freezing of gait episodes. During each training session, two video clips (with different sequences of actions) were presented twice. Another group of patients watched two video clips (presented twice) containing sequences of static pictures of mountains and seaside, countryside, and desert scenes without any living (human or animal) representations. Pelosin and colleagues observed that subjects submitted to video with active movements improved for freezing of gait better than patients who watched “static” video [10], even if it is also as yet not clear whether it would be more effective for movement execution to be carried out simultaneously, or following AOT. A further meaningful result has been obtained for the reduction of bradykinesia after only one session of AOT in PD patients compared to subjects submitted to acoustic cue [28]. To the best of our knowledge, to date, no studies have investigated the role of AOT in reducing balance and postural disorders in PD patients.

The pathogenesis of PD-related balance disorders and postural instability is likely multifactorial: dystonia, rigidity, proprioceptive and sensorimotor disintegration, and peripheral degenerative processes have been proposed as causative factors. Therefore, it is possible that lesions in nondopaminergic systems can play a role in the pathophysiology of postural instability in PD [27]. Strategies exercises classically employed for improving balance include external forces against which to perform voluntary movement (and neuromuscular responses) as well as in response to an unexpected perturbation/stimulus in order to maintain the body's centre of mass within manageable limits of the base of support or in transit to a new base of support. Between several techniques of rehabilitation, global postural rehabilitation, functional static and dynamic standing balance training, computerized balance training using visual feedback, strengthening exercises, dance, yoga, vibration platform, Tai Chi, and sensorial cues can exert physiological effects at the cortical level, acting on intracortical inhibition or excitation in the areas controlling the flexor and extensor muscles [29]. A recent study showed that four-week indoor training and four-week outdoor rehabilitation are sufficient to improve balance and posture in PD patients [30]. The authors used postural reeducation, flexibility exercises, strength training with functional tasks, balance dance, modified Wing Chun (Chinese martial art), and Square Stepping Exercise with eight special patterns with progressive difficulty levels aiming at multidirectional balance and gait skills [30]. Another study showed that twelve weeks of rehabilitative training focused on progressive exercises targeting improvements in the function of the deeper trunk muscles were effective in improving clinical measures of balance in people with PD [31]. Moreover, similar exercises, when combined with aerobic exercises and stretching, were shown to significantly improve the strength and mobility of the trunk muscles in individuals with PD. The combination of postural rehabilitation and Kinesio taping on trunk muscles, in a rehabilitative programme applied for four weeks, can be used for reducing axial postural disorders and risk of fall in PD [32].

The variability of the findings obtained in rehabilitative programmes and procedures confirms the absence of standard procedures to improve balance and posture in subjects affected by PD. In fact, the effect of several techniques is directed on impaired postural control systems, including flexibility and strength, anticipatory postural adjustment, postural responses sensory orientation, and stability in gait [33]. The purpose of the present study was to evaluate the effectiveness of AOT to reduce balance and postural disorders in PD patients. In the present study, the choice of the type of movements and therefore the content of the video to show were the result of numerous evaluations extrapolated from existing literature and also adapted to our population of PD patients. The motor sequences contained in the videos concerned the assumption of positions strictly linked to equilibrium when the PD patients are sitting, or when they are standing during ambulation. We focused on postural instability evaluated primarily with the BBS and treated with the AOT, a therapy that has been successfully integrated in numerous fields of neurorehabilitation. Hence, PD patients did not undergo traditional physical training programmes during the period of video-therapy training to avoid possible confounders examining possible effects of the AOT. To date, there is no evidence that AOT may be useful for reducing balance and postural disorders in PD patients. Our findings did not support the hypothesis to use this rehabilitative technique for this field. In fact, the analysis of outcome measures used to test patients at baseline and after two months of treatment showed only a slight improvement of clinical features of patients concerning balance and posture. The surprising effect of AOT in reducing freezing of gait episodes and bradykinesia [10, 28], as well as the recovery of the arm motricity in stroke survivors [7, 8], proved unsuccessful in reducing balance and postural disorders.

AOT is based on recruiting motor areas not only when actions are actually performed, but also when they are mentally rehearsed or simply observed. Mirror neurons are localized into premotor cortex and the adjacent area 44, the human homologue of area F5 [34, 35], first described in monkeys, so, they act on motor system improving upper or lower limb motricity. This could explain the reasons why our data did not significantly change after 8 weeks of AOT. Motor system is only partially involved in posture and balance control. It is a multifactorial process based on proprioceptive information (tactile, somatosensory, visual, and vestibular feedback) that by afferent pathways can modify efferent responses mediated by cerebellum and spinal cord on antigravitary muscles influencing muscle spindles, Golgi tendon organs, subcutaneous, somatosensory, and mechanoreceptors activity, and joints' position. All these components are involved to plan, organize, adjust, and execute postural and voluntary movements. Despite the possibility to increase the motor performances of PD patients submitted to our video for 8 weeks, our subjects did not report a statistically significant improvement of balance impairment and postural disorders measuring with primary and secondary outcome measures. This result is, moreover, in accordance with the conclusion of a review article on the role of motor learning in PD [36]. Nieuwboer and colleagues argued that PD patients do seem to need more time to achieve learning, especially to achieve automatization, and, especially in the later stages of the disease, explicit learning methods, sensory information, and cues may be adopted to enhance learning [36]. However, even against the background of a neurodegenerative condition as PD affecting the basal ganglia, animal models of PD suggested that there is a dynamic interplay between degenerative and regenerative mechanisms of these structures, which are mediated by exercise and learning. Focused physical activity may tap into a variety of molecular repair mechanisms which not only appear to restore motor function but also promote neuroprotection at least in PD animal models [36].

## 5. Conclusion

Despite the growing evidence on the utilization of AOT in the field of neurorehabilitation, in the present study, no positive evidence was found in improving balance and posture related disorders in mild to moderate PD patients in 8-week training sessions. We must acknowledge some limitations of the present study. First of all, the small sample size and the possibility of including other forms of degenerative parkinsonism despite our accuracy could impact the obtained findings. Probably, more targeted and task-specific activities of the AOT programmes (i.e., daily actions divided into the component activities) would be required to achieve an effect on postural instability in PD patients. Furthermore, no long-term follow-up was considered. Again, no instrumental evaluation of balance skills, such as computerized static and dynamic posturography, was done and no cued training was applied to the participants. Finally, given the absence of a standardized protocol, about video and dynamic images for AOT, no firm conclusions concerning the efficacy of AOT in PD patients to reduce balance and postural impairment could be drawn. Although this was a feasibility study with a relatively low sample size, the outcomes and the data are still important to report and lend valuable information to the field. Future efforts will strive to refine processes and approaches, based on these results and experiences. Therefore, further studies with larger samples, longer follow-up period, and standardized protocols with AOT only or plus traditional physiotherapy are needed to investigate the effects of this rehabilitation technique to manage postural and balance disorders in PD patients.

## Figures and Tables

**Table 1:** Videos for Action observation treatment (AOT) shown to the Parkinson's disease patients in the present open-label feasibility prospective study.

First 4-week AOT program	Last 4-week AOT program
*Unsupported Sitting on The Table with Upper Limbs Abducted to 90*°. The abduction movement is performed slowly by the actor, remaining for a few seconds with the shoulders abducted to 45° and subsequently completing the articular excursion up to 90°. The position is held for 90 seconds	*Unsupported Standing with One Foot for at Least 5 Seconds and Then Reverse*. The actor shifts his weight first on one foot and then on the other. The foot is kept away from the floor variably from patient to patient, at least 5 seconds. Where necessary, he may lean on the wall with the contralateral hand

*Picking Something off the Floor*. In the first part of the video, the actor is standing as close as possible to the object; then, bending one knee on the floor surface, he picks it up. After collecting the first object, again from standing position, he flexes the contralateral knee to pick up the second object	*Walking Sideways*. The exercise is done without sliding foot or twisting body. The actor stands with feet close together. Then, stepping sideways, he moves one feet to the side first and brings the contralateral to join it

*Walking in a Straight Path with Upper Limbs Abducted at 90*°. Initially, the actor walks back and forth with arms at his sides. The following sequence requires shoulder abduction to 90° wherever possible. Additional external cues, such as marked path, were avoided	*Overcoming an Obstacle (Tightrope to 15 cm from the Floor)*. The actor is standing next to a tightrope to 15* *cm (at least) from the floor. Firstly, he lifts up one feet placing it over the rope; then, he brings the opposite feet to join it. Subsequently, twisting his body, he returns to start position

*Walking in a Not Straight Path with Upper Limbs Abducted at 90*°. The actor walks on a curved path, first with his arms at his sides and then with shoulders abducted up to maximum 90°. The patient is asked to walk so that he draws an imaginary “8” on the floor	*Getting Round Obstacles (Pins)*. The pins are placed in series across the floor at distances of 40* *cm. The actor walks through them trying to avoiding them. Arrived at the last one pin, he turns around to restart the exercise

**Table 2:** Demographical and clinical characteristics of 15 patients with Parkinson's disease (PD) enrolled. Data are presented as mean ± standard deviation (SD) or percentage.

Gender	
Female *n* (%)	9 (60)
Male *n* (%)	5 (40)
Age (years) mean ± SD	68.9 ± 4.7
Time from idiopathic PD diagnosis (years) mean ± SD	3.5 ± 1.9
H&Y score mean ± SD	2.1 ± 0.8
More affected body side (right/left)	10/5
FAC mean ± SD	4.3 ± 0.5
MMSE mean ± SD	26.4 ± 1.45

H&Y: Hoehn & Yahr stage; FAC: Functional Ambulation Category; and MMSE: Minimental State Examination.

**Table 3:** Functional findings of 15 patients with Parkinson's disease at baseline (*t*_0_) and at the end of the 8-week program of action observation treatment (AOT) (*t*_1_). Data are presented as mean ± standard deviation (SD) and 95% confidence interval (95% CI).

Outcome measures	*t*_0_	95% CI	*t*_1_	95% CI	Score differences	*p* value
BBS	42.9 ± 6.9	39.0–46.7	44.8 ± 7.0	40.9–48.7	−1.93	0.3706
ABC-16	49.8 ± 13.8	42.2–57.5	51.9 ± 13.9	44.2–59.6	−2.09	0.5201
10MWT (s)	16.1 ± 3.3	14.2–17.9	15.0 ± 3.3	13.2–16.9	1.04	0.1645
TUGo (s)	15.3 ± 4.2	12.9–17.6	14.1 ± 4.1	11.8–16.3	1.21	0.3613
UPDRS III	23 ± 13.1	15.7–30.2	22.1 ± 13.0	14.8–29.2	0.93	0.8031

95% CI: 95% confidence interval; BBS: Berg Balance Scale; ABC-16: Activities-Specific Balance Confidence Scale; 10MWT: 10-Meter Walk Test; TUG: Timed Up and Go Test; UPDRS III: Unified Parkinson's Disease Rating Scale, part III.
